# Coronary calcium scoring on virtual non-contrast and virtual non-iodine reconstructions compared to true non-contrast images using photon-counting computed tomography

**DOI:** 10.1007/s00330-023-10402-y

**Published:** 2023-11-09

**Authors:** Simran P. Sharma, Judith van der Bie, Marcel van Straten, Alexander Hirsch, Daniel Bos, Marcel L. Dijkshoorn, Ronald Booij, Ricardo P. J. Budde

**Affiliations:** 1 Department of Radiology & Nuclear Medicine, Erasmus MC, University Medical Center Rotterdam, Rotterdam, The Netherlands; 2 Department of Cardiology, Erasmus MC, University Medical Center Rotterdam, Rotterdam, The Netherlands; 3 Department of Epidemiology, Erasmus MC, University Medical Center Rotterdam, Rotterdam, The Netherlands

**Keywords:** Coronary artery disease, Computed tomography angiography, Image processing (Computer-assisted)

## Abstract

**Objectives:**

To compare coronary artery calcification (CAC) scores measured on virtual non-contrast (VNC) and virtual non-iodine (VNI) reconstructions computed from coronary computed tomography angiography (CCTA) using photon-counting computed tomography (PCCT) to true non-contrast (TNC) images.

**Methods:**

We included 88 patients (mean age = 59 years ± 13.5, 69% male) who underwent a TNC coronary calcium scan followed by CCTA on PCCT. VNC images were reconstructed in 87 patients and VNI in 88 patients by virtually removing iodine from the CCTA images. For all reconstructions, CAC scores were determined, and patients were classified into risk categories. The overall agreement of the reconstructions was analyzed by Bland–Altman plots and the level of matching classifications.

**Results:**

The median CAC score on TNC was 27.8 [0–360.4] compared to 8.5 [0.2–101.6] (*p* < 0.001) on VNC and 72.2 [1.3–398.8] (*p* < 0.001) on VNI. Bland–Altman plots depicted a bias of 148.8 (ICC = 0.82, *p* < 0.001) and − 57.7 (ICC = 0.95, *p* < 0.001) for VNC and VNI, respectively. Of all patients with CAC_TNC_ = 0, VNC reconstructions scored 63% of the patients correctly, while VNI scored 54% correctly. Of the patients with CAC_TNC_ > 0, VNC and VNI reconstructions detected the presence of coronary calcium in 90% and 92% of the patients. CAC_VNC_ tended to underestimate CAC score, whereas CAC_VNI_ overestimated, especially in the lower risk categories. According to the risk categories, VNC misclassified 55% of the patients, while VNI misclassified only 32%.

**Conclusion:**

Compared to TNC images, VNC underestimated and VNI overestimated the actual CAC scores. VNI reconstructions quantify and classify coronary calcification scores more accurately than VNC reconstructions.

**Clinical relevance statement:**

Photon-counting CT enables spectral imaging, which might obviate the need for non-contrast enhanced coronary calcium scoring, but optimization is necessary for the clinical implementation of the algorithms.

**Key Points:**

• *Photon-counting computed tomography uses spectral information to virtually remove the signal of contrast agents from contrast-enhanced scans*.

• *Virtual non-contrast reconstructions tend to underestimate coronary artery calcium scores compared to true non-contrast images, while virtual non-iodine reconstructions tend to overestimate the calcium scores*.

• *Virtual non-iodine reconstructions might obviate the need for non-contrast enhanced calcium scoring, but optimization is necessary for the clinical implementation of the algorithms*.

## Introduction

Coronary artery calcification (CAC) is a marker of coronary atherosclerosis and a predictor of cardiovascular events [1, 2]. The absence of coronary calcium is associated with a very low prevalence of obstructive cardiovascular disease (CAD) and a very low risk of death or non-fatal myocardial infarction [2]. CAC is often quantified using the Agatston scoring method on non-enhanced CT scans [3], and a higher CAC score is associated with increased probability of cardiovascular events [4].

Coronary CT angiography (CCTA) is needed to assess the coronary lumen and the presence and degree of coronary stenosis. However, it is challenging to calculate CAC scores on CCTA images due to overlap of the CT numbers of iodine contrast-enhanced blood and calcium [5]. As a result, a non-enhanced scan is often obtained in addition to CCTA to calculate CAC scores in clinical practice.

Photon-counting computed tomography (PCCT), which uses photon-counting detectors (PCDs) instead of energy-integrating detectors (EIDs), has recently become available for clinical use. PCCT has the potential to address various limitations of current CT technology and offers a higher spatial resolution due to its higher geometric detector efficiency. [6] It also enables spectral reconstruction techniques which improves material decomposition and allows for exclusion of electronic noise, multi-contrast agent imaging, and spectral reconstructions such as virtual-mono-energetic images. With dual-source PCD CT, high temporal resolution spectral imaging becomes feasible [6, 7].

Reconstruction algorithms like virtual non-contrast (VNC) and virtual non-iodine (VNI) create images by virtually removing iodine from the CCTA without additional scanning. For dual-energy CT (DECT) imaging, VNC reconstructions have been available for a decade. Virtual non-iodine is a novel reconstruction algorithm specifically designed to depict and quantify calcifications on PCCT angiography images. These reconstructions have the potential to replace true non-contrast (TNC) scans, which could eventually lead to dose reduction.

Research about the performance of these two algorithms is still limited [8, 9]. To date, only two studies have examined VNC and/or VNI for CAC scoring using a photon-counting system in a clinical set-up [9, 10]. However, it is important to note that one of these studies was performed with an older software version on a dedicated research workstation [9], while the other study did not analyze the calcium scores using the standard reconstruction parameters for the Agatston method [10]. Our aim was to evaluate the performance of VNC and VNI obtained from CCTA scans to detect and quantify coronary calcium, using the CAC score of TNC scans as the reference standard, on a commercially available dual-source PCCT scanner.

## Materials and methods

### Study population

All consecutive adult patients (aged > 18 years) who underwent a CCTA on a dual-source PCCT scanner (Siemens NAEOTOM Alpha, Siemens Healthineers VA50A) as part of clinical care in our centre between January 24, 2022 and July 16, 2022, and had a TNC scan and VNC/VNI reconstructions, were included in the study. Written informed consent was obtained from all patients. The study was performed in line with the principles of the Declaration of Helsinki. Since this is a purely observational and retrospective study, the need for ethics committee approval was waived by the institutional review board.

### Image acquisition and reconstruction

First, a true non-contrast acquisition was performed using the following acquisition parameters: prospective ECG-triggering, tube voltage 120 kV, collimation of 144 × 0.4 mm, and 0.25-s rotation time. All TNC images were acquired with an image quality level setting of 16. TNC images were reconstructed at 70 keV with 3-mm slice thickness, 1.5-mm increments, kernel Qr36, and an iterative reconstruction strength of 3.

Subsequently, a contrast-enhanced scan of the heart was performed. The CCTA acquisition protocol was chosen in accordance with the clinical question. All CCTAs were acquired at 120 kV and using prospective ECG-triggering. The tube current–time product was automatically adjusted to achieve an image quality level of 65 for CCTA and 34 for transcatheter aortic valve implantation (TAVI) planning scans.

VNC and VNI reconstructions were derived from the contrast-enhanced scans. To ensure comparability, we matched the image reconstruction parameters for VNC and VNI with TNC. All VNC and VNI images were reconstructed at the scanner at 70 keV with 3.0-mm slice thickness, 1.5-mm increments, and Qr36 kernel (Syngio.via version VA50A.2.03).

**Fig. 1:**
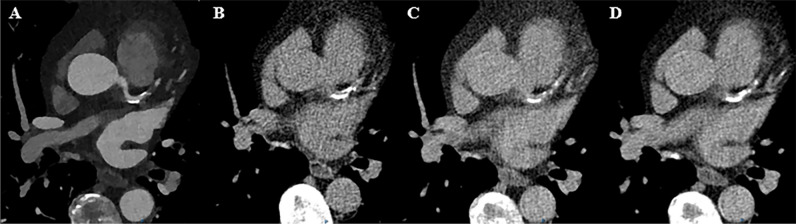
Calcium scoring from the photon-counting CT for a patient with calcifications in the LAD. **A** Contrast-enhanced CCTA. **B** Standard non-contrast imaging for calcium scoring (CAC = 461.1). **C** Virtual non-contrast reconstruction (CAC = 179.5). **D** Virtual non-iodine reconstruction (CAC = 538)

VNC images are reconstructed by material decomposition of water and iodine and will artificially decompose calcium into these two components. In general, the material attenuation curves of the known materials, in this case water and iodine, are used to represent another material by a linear combination [3, 11, 12]. The spectral separation in PCCT allows the attenuation measurements to be divided into low energy and high energy bins, creating two virtual energy levels which are used for material decomposition to create VNC images [8, 13]. The VNI algorithm (PureCalcium, Siemens Healthineers) performs a series of routines to subtract iodine and preserve calcium. In the detection step, based on the spectral properties of calcium and iodine, a ‘non-calcium mask’ is generated. This mask helps to preserve full calcium contrast for the selected mono-energetic reconstruction [9]. Figure 1 displays the CAC scoring on PCCT images.

### Assessment of the CAC scores

**Fig. 2:**
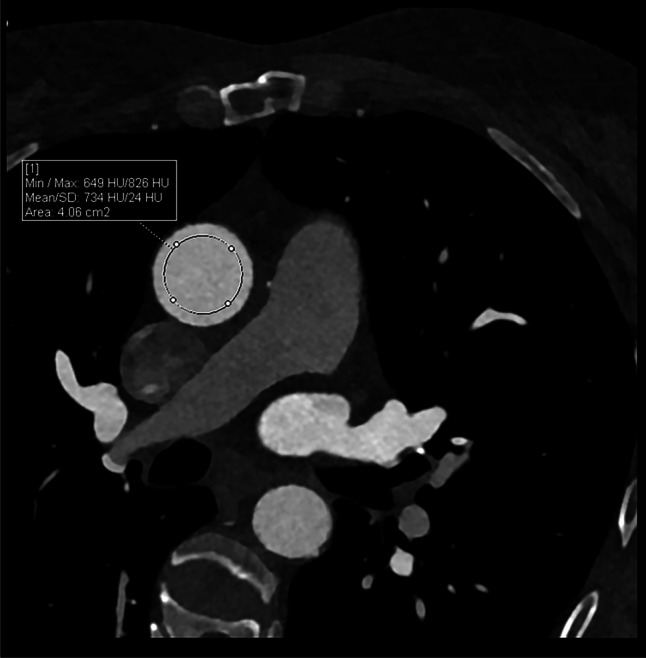
Iodine attenuation measurement. A region-of-interest was placed in the ascending aorta. CT numbers of the region-of-interest together with the standard deviation (noise) were automatically measured

All image analyses were performed using commercially available software (Syngo.via, version VB60Av; Siemens Healthineers). Two researchers, with 2 years of experience in cardiovascular CT quantified the CAC scores on the TNC, VNC, and VNI images by using the semi-automatic calcium scoring assessment. Both researchers worked together to grade the cases and were supervised by an experienced radiologist with over 15 years of experience in cardiovascular CT. The calcium scoring algorithm uses a Hounsfield unit (HU) threshold of 130 with connected component analysis (≥ 1 mm^2^) to classify the detected voxels as calcifications [3]. Additionally, the volume of the calcifications was computed. Patients were subsequently classified by CAC_TNC_ in the following categories expressed in Agatston scores: none (CAC = 0), minimal (CAC > 0–10), mild (CAC > 10–100), moderate (> 100–400), and severe (CAC > 400) [14]. To determine the influence of contrast density on the CAC score, a circular region-of-interest (ROI) was drawn as large as possible in the ascending aorta on the CCTA 55-keV images. From the ROIs, the mean CT number and standard deviation of the iodine contrast agent were derived for comparison between iodine attenuation values and calculated CAC scores (Fig. 2).

### Statistical analysis

Categorical variables are expressed as frequencies and percentages, continuous variables were expressed as mean ± standard deviation or as median with inter-quartile range. To compare the CAC scores from the VNC and VNI sequences with TNC, we followed the following strategy. Median CAC scores and median volumes of all reconstructions were compared by the Wilcoxon signed rank test. Next, we assessed the agreement of TNC with VNC and VNI using Bland–Altman analyses and calculated intra-class coefficients (ICCs) for TNC with VNC and VNI. We aimed to quantify the differences between the presence/absence of CAC on TNC with VNC and VNI, including the sensitivity, specificity, and positive and negative predictive values. The categorical CAC_TNC_ was compared to the categorical CAC_VNC_ and CAC_VNI_ to evaluate if the spectral reconstructions would classify the patients in the proper category. The correlation between mean HU values of iodine contrast on CCTA images and CAC score on VNC and VNI was assessed using a Pearson correlation coefficient (*r*). All statistical analyses were performed using SPSS statistical software (IBM Corp. Released 2016. IBM SPSS Statistics for Windows, version 28.0.1.0) and Python (Python Software Foundation. Python Language Reference, version 3.9).

## Results

### Study population

In total, 88 patients (mean age 59 years, SD 13.5; 61 men (69%)) were included. For one patient, VNC images were not reconstructed due to unknown reasons. The indication for scanning was evaluation of CAD (*n* = 70), pulmonary vein ablation planning (*n* = 8), TAVI planning (*n* = 4), evaluation of pericardial effusion (*n* = 2), evaluation of coronary anomalies (*n* = 1), exclusion of left atrial/ventricular thrombus (*n* = 2), and evaluation of malignancy (*n* = 1). The median CTDI_vol_ for TNC was 2.3 mGy [1.6–2.9] and for CCTA 14.6 mGy [10.4–23.2]. The patient characteristics, scan protocols, and radiation dose are presented in Table 1.

**Table 1:** Baseline study characteristics

Patient characteristics	
Male (%)	61 (69.3)
Age	59 (13.5)
BMI	27 (4.9)
PCI in history (%)	6 (6.8)
Scan protocol	
Prospective trigger with relative systolic ECG pulsing (%)	32 (36.4)
Prospective trigger with relative diastolic ECG pulsing (%)	29 (33.0)
Prospective trigger with relative systolic and diastolic ECG pulsing (%)	12 (13.6)
Ultra-fast, low dose high pitch (%)	7 (8.0)
Prospective trigger with relative systolic ECG pulsing (using forced maximum collimation setting) (%)	4 (4.5)
Prospective trigger with absolute systolic ECG pulsing (%)	2 (2.3)
Prospective triggered high pitch spiral (%)	2 (2.3)
CT radiation dose	
TNC DLP, mGy*cm	31.7 [25.2–39.7]
TNC CTDI_vol_, mGy	2.3 [1.6–2.9]
TNC effective mAs	16.0 [13.0–20.0]
CCTA DLP, mGy*cm	190.0 [129.0–297.0]
CCTA CTDI_vol_, mGy	14.6 [10.4–23.2]
CCTA effective mAs	38.0 [32.0–50.0]

Data is presented as mean ± standard deviation (SD), median (25th–75th percentile), or frequencies (percentage). *BMI*, body mass index; *ECG*, electrocardiogram; *PCI*, percutaneous coronary intervention; *PC*, pure calcium; *TNC*, true non-contrast; *VNC*, virtual non-contrast; *CTDI*_*vol*_, CT dose index

### Calcium scoring and volumes

**Fig. 3:**
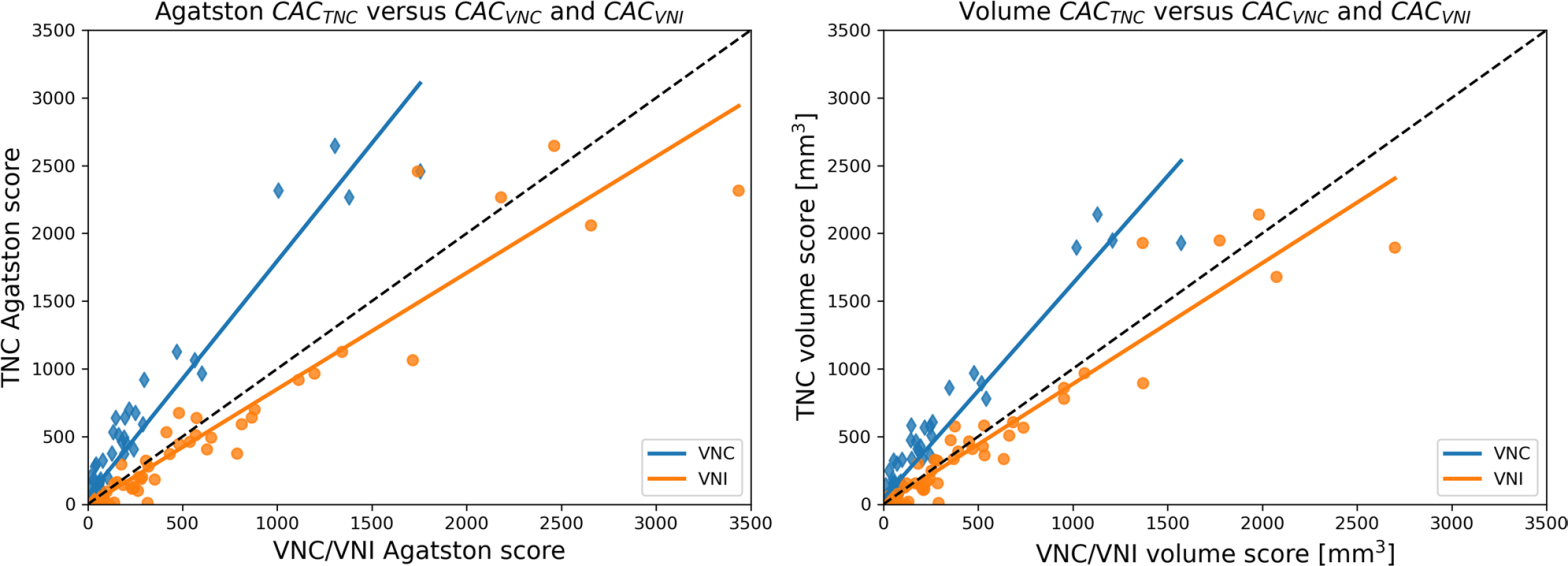
Comparison of VNC and VNI calcium scores and volumes with the TNC calcium scores and volumes. The scatterplots indicate a general overestimation of the VNI values (orange dots) compared to the TNC values for both the CAC scores and volumes. The VNC scores (blue dots) show a consistent underestimation of the CAC scores and volumes compared to TNC

The median CAC score on TNC was 27.8 [0–360.4], on VNC reconstructions it was significantly lower (8.5 [0.2–101.6]; *p* < 0.001), while the CAC score on VNI reconstructions was significantly higher (72.2 [1.3–398.8]; *p* < 0.001). Compared to the median CAC volumes on TNC, the CAC volumes were significantly lower on VNC and higher on the VNI reconstructions (Table 2). Figure 3 illustrates the VNC and VNI scores and volumes plotted against the values of TNC.

**Table 2:** Comparison of CACs and CAC volumes of TNC, VNC, and VNI reconstructions

	Median	Median TNC	*p*
CAC score			
VNC	8.5 [0.2–101.6]	27.8 [0–360.4]	< 0.001
VNI	72.2 [1.3–398.8]	27.8 [0–360.4]	< 0.001
CAC volume			
VNC	16.0 [0.1–98.3]	34.1 [0–333.0]	< 0.001
VNI	69.1 [2.6–337.8]	34.1 [0–333.0]	0.002

Data is presented as median (25th–75th percentile). *PC*, pure calcium; *CAC*, coronary artery calcium; *TNC*, true non-contrast; *VNC*, virtual non-contrast; *VNI*, virtual non-iodine

### Bland–Altman analysis

**Fig. 4:**
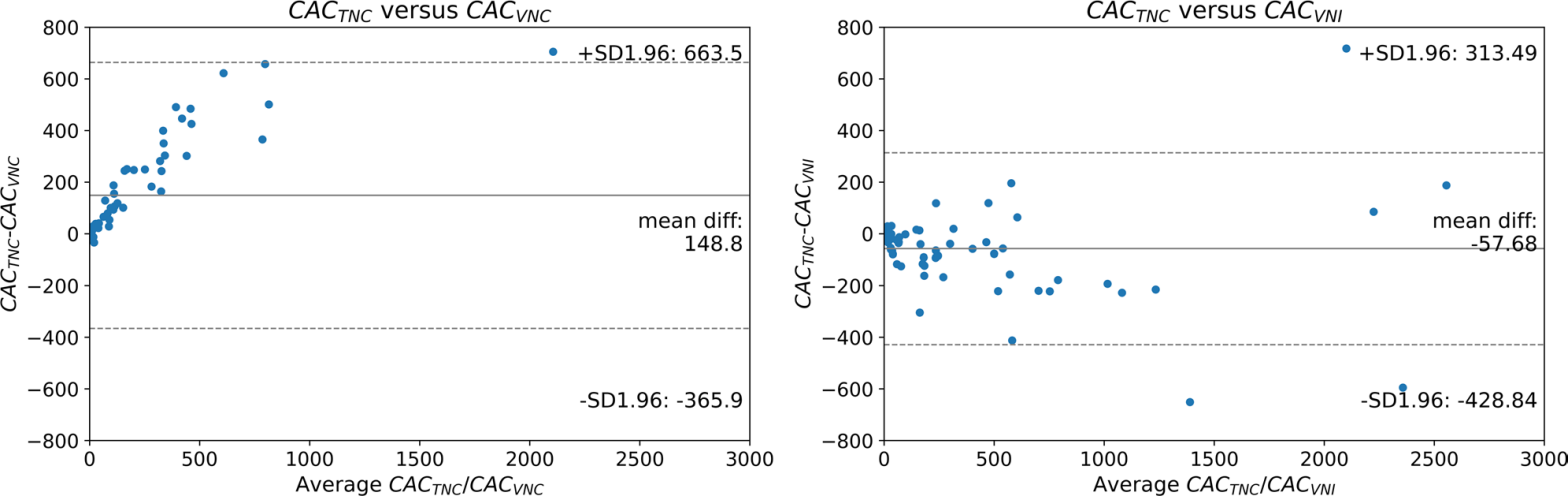
Bland–Altman plots. The difference between the TNC score and VNC/VNI is displayed on the vertical axis. On the *x*-axis, the mean of the two measurements is plotted. VNC shows an underestimation of the CAC scores compared to TNC. VNI shows an overestimation compared to TNC.

Bland–Altman analysis of CAC_TNC_ versus CAC_VNC_ and CAC_VNI_ are displayed in Fig. 4. The plots show a mean bias of 148.8 (ICC = 0.82, *p* < 0.001) related to an underestimation of VNC scores compared to TNC scores. In addition, lower scores appear to have less variation than higher scores. For the VNI reconstructions, an overall bias of − 57.7 (ICC = 0.95, *p* < 0.001) was observed, indicating smaller bias compared to VNC. In addition, no clear systematic error was found for the VNI reconstructions.

### Presence or absence of coronary calcifications

Table 3 shows that on the TNC images, 24 patients had a CAC score of zero and 64 patients a CAC score greater than zero. Of the 24 patients with a CAC score of zero on the TNC reconstructions, 15 (63%) had a CAC score of zero on the VNC reconstructions and 13 (54%) on the VNI reconstructions. In case of a CAC score of zero on TNC, the median CAC score on the VNC reconstructions (excluding patients with a score of zero) was 2.6 [0.9–3.3] and on the VNI reconstructions (excluding patients with a score of zero) 19.4 [13.6–61.5]. Of the 64 patients with a CAC score greater than zero on the TNC reconstructions, 57 (90%) also scored greater than zero on the VNC reconstructions and 59 (92%) on the VNI reconstructions. The sensitivity, specificity, and positive and negative predictive values for VNC were 90%, 63%, 86%, and 71% and for VNI were 92%, 54%, 84%, and 72%. The area under the curve (AUC) for VNC and VNI to detect coronary calcium using TNC as the reference standard were 0.88 (95% CI: 0.82–0.85) and 0.87 (95% CI: 0.80–0.94), respectively.

**Table 3:** CAC scores based on TNC (rows) and VNC/VNI (columns)

CAC_VNC_							
		None	Minimal	Mild	Moderate	Severe	Total
CAC_TNC_	None	**15**	8	1	0	0	24
	Minimal	3	**8**	1	0	0	12
	Mild	3	7	**6**	0	0	16
	Moderate	0	1	12	**3**	0	16
	Severe	0	0	0	12	**7**	20
	Total	21	24	20	15	7	
CAC_VNI_							
CAC_TNC_	None	**13**	2	8	1	0	24
	Minimal	2	**4**	6	0	0	12
	Mild	3	4	**9**	0	0	16
	Moderate	0	0	0	**14**	2	16
	Severe	0	0	0	0	**20**	20
	Total	18	10	23	15	22	

### Reclassification percentages

**Fig. 5:**
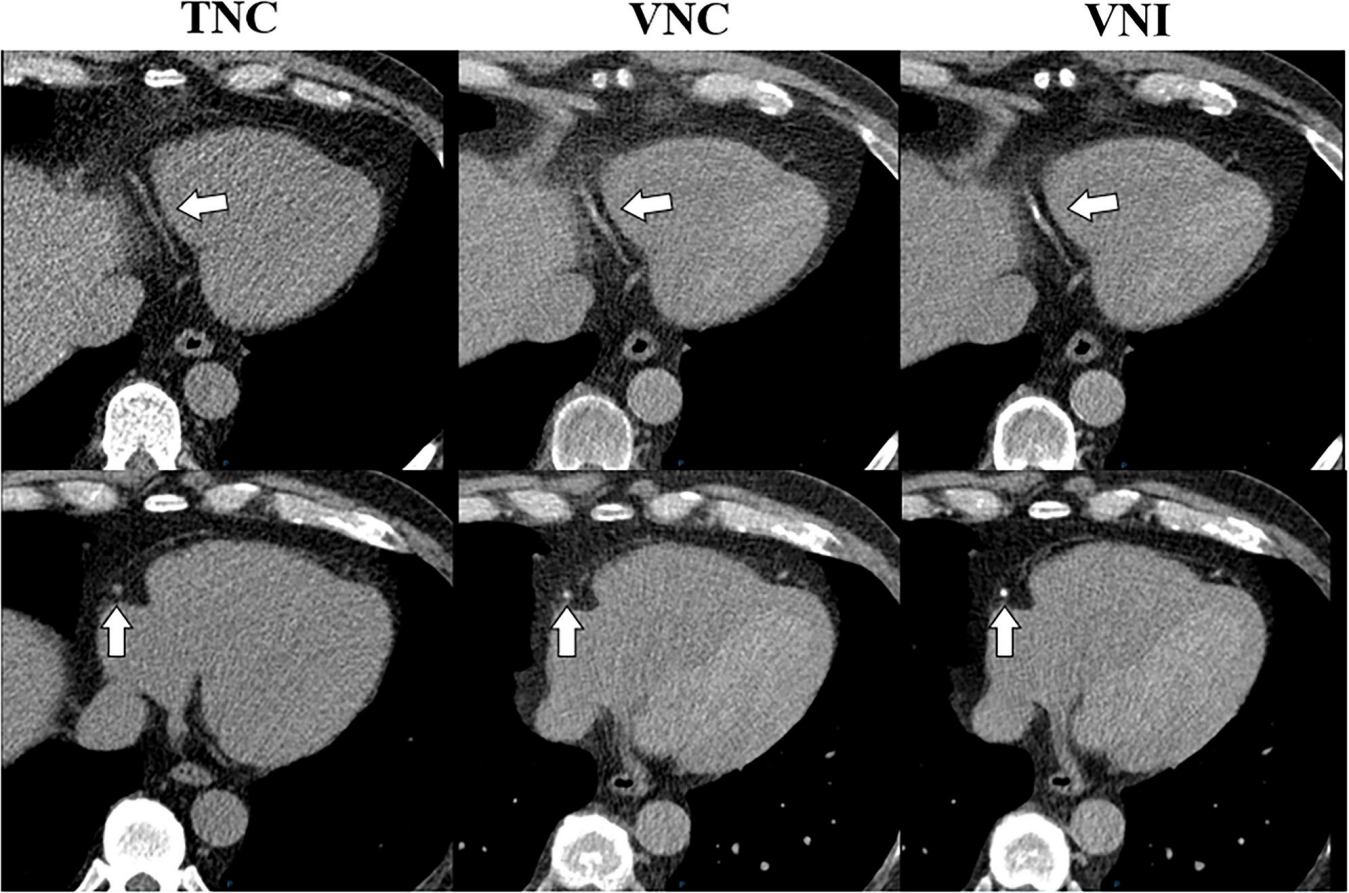
Calcium scoring on TNC, VNC, and VNI in the right coronary artery. A 63-year-old male patient suspected of coronary artery disease underwent calcium scoring and CTA. No calcifications in the RCA were present on the TNC images, while calcifications were detected on both VNC and VNI

In total, 55% (48/87) of the patients were misclassified by the VNC reconstructions and 32% (28/88) by the VNI reconstructions. We observed that in moderate and severe CAC scores, the VNC algorithm misclassified 25 of the 35 patients (71%), while the VNI algorithm only misclassified two patients of the 36 patients (6%). The VNC algorithm has fewer misclassifications in lower-risk groups (none, minimal) while VNI performs better in higher-risk groups (mild, moderate, and severe) in terms of classification. Figure 5 shows an example case with absence of CAC on TNC, but presence of CAC on VNC and VNI.

### HU values of iodine in CCTA scans

**Fig. 6:**
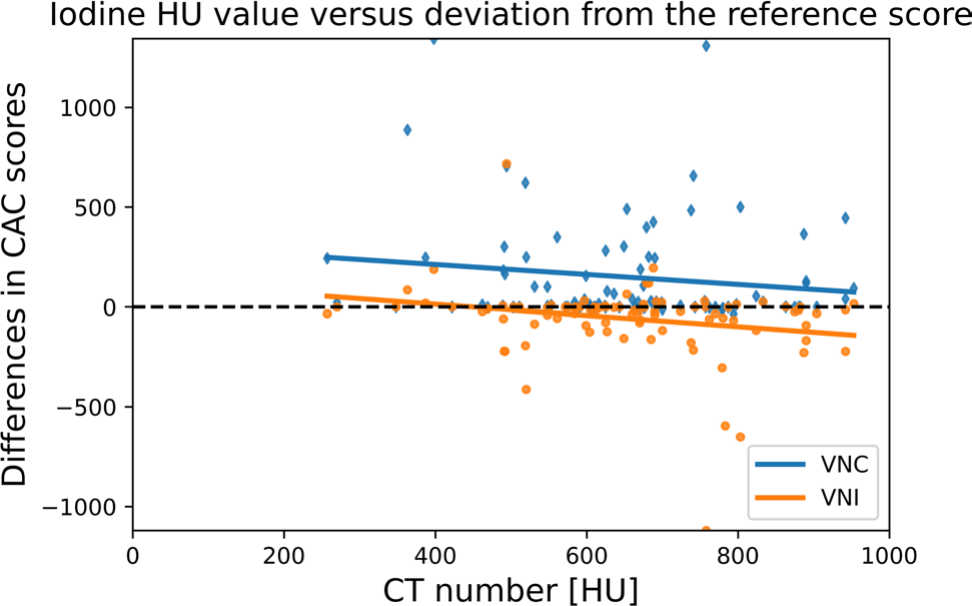
Mean iodine CT numbers measure on 55-keV CCTA images plotted against CAC scores. The values on the *x*-axis represent the mean iodine HU values, and those on the *y*-axis the CAC scores. Values around the zero indicate no to limited differences between CAC scores. The figure also includes outliers for CAC scores over the entire range of iodine HU values, indicating that there is no clear relationship between iodine HU values and the accuracy of CAC scores. A mean standard deviation of 30 HU was found

HU values of iodine in CCTA scans were plotted against the difference between CAC_TNC_ and CAC_VNC_ and difference between CAC_TNC_ and CAC_VNI_ to investigate whether this influenced the measurement errors. Figure 6 demonstrates a lack of correlation between iodine HU values and CAC scores (VNC; *r* =  − 0.16; *p* = 0.13 and VNI; *r* =  − 0.15; *p* = 0.16).

## Discussion

This study evaluated the performance of VNC and VNI reconstructions in PCCT using TNC images as reference. First, both VNC and VNI reconstructions are better at detecting the presence of coronary calcium than the absence of coronary calcium. Secondly, compared to TNC images, VNC scored lower median CAC scores and VNI scored higher median CAC scores. VNI reconstructions outperform VNC reconstructions regarding the accuracy of CAC quantification and classification. However, both VNC and VNI reconstructions currently seems to be not reliable enough to fully replace TNC acquisition for coronary calcium scoring.

Our study found that both VNC and VNI inaccurately estimate coronary artery calcium (CAC) scores, which can result in false negatives, false positives, and incorrect risk categorizations. The overestimation of CAC scores by VNI in the absence of CAC on TNC can impact clinical decision-making, potentially resulting in incorrect risk stratification and inappropriate treatment decisions, such as unnecessary additional testing for CAD [15]. However, validating the VNI results with the CCTA may aid in avoiding overestimation of the CAC score. Compared to VNC, VNI misclassified more patients with a CAC score of zero on TNC and less patients with a CAC score in the moderate and severe categories. On the other hand, VNC frequently misclassified patients with moderate or severe CAC scores as lower risk, potentially leading to inadequate treatment.

VNC reconstructions which are not specifically designed for CAC scoring have been available for a decade. Two studies that investigated the VNC reconstruction on dual-energy systems found a high correlation but systematic underestimation of CAC scores [5, 16]. The structural underestimation of CAC_VNC_ compared to CAC_TNC_ in these studies is in line with our findings and leaves the clinical application of VNC reconstructions for CAC scoring not forthcoming. Additionally, the Bland–Altman analyses in our study showed that VNI reconstructions exhibited a smaller bias compared to VNC reconstructions. On top of that, the absence of a clear systematic error in VNI reconstructions supports their higher accuracy in CAC scoring. These findings suggest that VNI reconstructions are potentially more appropriate than VNC reconstructions for CAC scoring.

Limited research is available about the performance of VNI reconstruction with clinical data. The study of Emrich et al concluded that VNI can accurately estimate CAC categories but significantly underestimated the CAC score [9]. However, in our study, we observed that VNI algorithm tended to overestimate the CAC score when compared to TNC images. These differing results may be attributed to variations in software version and the method of reconstructing the VNC/VNI images. Emrich et al utilized offline reconstructions performed on a dedicated research workstation, whereas our reconstructions were performed at the scanner. Besides that, we identified differences between our study design and population when compared to Emrich et al. Images were reconstructed at a higher iterative reconstruction strength in our study. We also observed that the median CAC scores on TNC were lower in our study population compared to population of Emrich et al. This difference may be attributed to the fact that only 4.5% (4/88) of our study population underwent CCTA for TAVI planning, while in the study by Emrich et al, 52.2% (35/67) of the population underwent CCTA for TAVI planning. This suggests that our study population and the population in the study by Emrich et al are significantly different, which may account for the lower median CAC scores in our population.

Fink et al investigated the effect of virtual mono-energetic images (VMIs) and QIR on the accuracy of coronary artery calcium scoring using VNI reconstructions [10]. The study found that decreasing VMI levels resulted in a consistent and significant increase in CAC scores, while the effect of QIR was less pronounced. Fink et al identified 55 keV or 60 keV as the optimal VMI levels. In our study, we reconstructed images at 70 keV since our research primarily focused on Agatston calcium scoring, which prescribes specific acquisitions and reconstruction parameter settings. Importantly, it should be noted that Fink et al excluded patients with a CAC score of zero, which differs from the approach adopted in our study.

When analysing the difference in the CAC scores between the reconstructions, inter-scan variability in CAC score quantification should be considered. In a large asymptomatic population, Yoon et al reported a difference between calcium scores in two consecutive scans of up to 28.4% in women and 43.0% in men [17]. As CAC_TNC_ and CAC_VNC_/CAC_VNI_ are derived from two separate scans, inter-scan variability might partly explain the differences in CAC scores. Other factors like heart rate and cardiac phase might also influence the CAC quantification [18, 19].

The Agatston score is based on a threshold method where pixels > 130 HU are considered calcifications [3]. Since VNC and VNI images are computed from the CCTA scans, the HU value of iodine could influence the CAC scores of these reconstructions. A recent phantom study concluded that VNC-based Agatston scores were similar in images with different in-vessel contrast densities (500 HU and 800 HU). Conversely, Agatston scores derived from VNI reconstructions were significantly higher at 500 HU than at 800 HU [20].

Nevertheless, in our study, no correlation was found between the amount of iodine present and the deviation of the CAC score from the ground truth.

One of the main strengths of this study is its large sample size and the range of CAC scores included; however, it also has several limitations. Firstly, the study was conducted at a single academic hospital, which may not be representative of the wider population. Furthermore, the effect of heart rate on the accuracy of the CAC scores measured with VNC and VNI reconstructions was not analyzed in this study. Recent insights from a phantom study demonstrated decreasing VNI-based CAC scores at rising heart rates; nevertheless, the VNI algorithm seemed to be less prone to cardiac motion in comparison to VNC [20]. One potential benefit of photon-counting CT might be the ability to detect smaller calcifications that may not have been visible with EID-CT due to its increased spatial resolution. However, the Agatston scoring method, which is used to evaluate images for the presence of calcifications, has a dated design that reconstructs images at a slice thickness of 3.0 mm. This negates the advantages of PCCT’s higher spatial resolution and hinders the use of improved reconstruction methods. To fully realise the benefits of PCCT, it may be necessary to revise the Agatston scoring method. Lastly, in this study, the scans were acquired at 120 kV; however, adjusting the tube voltage might improve the accuracy of the VNI algorithm.

In conclusion, compared to TNC images, both VNC and VNI reconstructions are better at identifying the presence of CAC than the absence of CAC. However, VNC tends to underestimate CAC scores and VNI tends to overestimate them. VNI reconstructions outperform VNC reconstructions regarding the accuracy of CAC quantification. In terms of risk categorisation, VNC misclassified 55% of the patients, while VNI misclassified 32%. Overall, these results suggest that neither VNC nor VNI reconstructions are currently accurate enough to fully replace TNC images for CAC scoring.

## Abbreviations

CAC: Coronary artery calcifications
PCCT: Photon-counting computed tomography
TNC: True non-contrast
VMI: Virtual mono-energetic images
VNC: Virtual non-contrast
VNI: Virtual non-iodine

## Declarations

### Guarantor

The scientific guarantor of this publication is prof. dr. Ricardo P.J. Budde.

### Statistics and biometry

Prof. dr. D. Bos kindly provided statistical advice for this manuscript.

### Methodology

• retrospective

• observational

• performed at one institution
